# CNS Invasion in Meningioma—How the Intraoperative Assessment Can Improve the Prognostic Evaluation of Tumor Recurrence

**DOI:** 10.3390/cancers12123620

**Published:** 2020-12-03

**Authors:** Felix Behling, Christina Fodi, Irina Gepfner-Tuma, Kathrin Machetanz, Mirjam Renovanz, Marco Skardelly, Antje Bornemann, Jürgen Honegger, Ghazaleh Tabatabai, Marcos Tatagiba, Jens Schittenhelm

**Affiliations:** 1 Department of Neurosurgery, University Hospital Tübingen, Eberhard-Karls-University Tübingen, 72076 Tübingen, Germany; christina-katharina.fodi@med.uni-tuebingen.de (C.F.); kathrin.machetanz@med.uni-tuebingen.de (K.M.); mirjam.renovanz@med.uni-tuebingen.de (M.R.); marco.skardelly@med.uni-tuebingen.de (M.S.); juergen.honegger@med.uni-tuebingen.de (J.H.); ghazaleh.tabatabai@med.uni-tuebingen.de (G.T.); marcos.tatagiba@med.uni-tuebingen.de (M.T.); 2 Center for CNS Tumors, Comprehensive Cancer Center Tübingen-Stuttgart, University Hospital Tübingen, Eberhard-Karls-University Tübingen, 72076 Tübingen, Germany; irina.gepfner-tuma@med.uni-jena.de (I.G.-T.); antje.bornemann@med.uni-tuebingen.de (A.B.); jens.schittenhelm@med.uni-tuebingen.de (J.S.); 3 Department of Neurology, University Hospital Tübingen, Eberhard-Karls-University Tübingen, 72076 Tübingen, Germany; 4 Interdisciplinary Division of Neuro-Oncology, University Hospital Tübingen, Eberhard-Karls-University Tübingen, 72076 Tübingen, Germany; 5 Hertie Institute for Clinical Brain Research, 72076 Tübingen, Germany; 6 Department of Neuropathology, University Hospital Tübingen, Eberhard-Karls-University Tübingen, 72076 Tübingen, Germany; 7 German Cancer Consortium (DKTK), DKFZ Partner Site Tübingen, 72076 Tübinen, Germany

**Keywords:** meningioma, brain invasion, CNS invasion, invasive growth, intraoperative assessment, recurrence risk, progression-free survival

## Abstract

**Simple Summary:**

Brain invasion has been integrated into the new WHO classification of meningiomas to improve the prognostic assessment regarding tumor recurrence. However, its role has been questioned. One of the reasons is that for complete histopathological assessment, tissue sampling of the complete brain–tumor interface is necessary, but not always surgically and technically feasible. Therefore, the additional intraoperative assessment of CNS invasion may be of value for a more precise assessment of this tumor characteristic. We therefore studied the prognostic impact of the histopathological and intraoperative assessment of CNS invasion regarding radiographic tumor recurrence and found that both factors by themselves do not reach a prognostic significance. However, if both factors are combined, CNS invasion is an independent negative prognostic factor. Our findings show the prognostic potential of a thorough assessment and underline the need for a standardization and documentation of meningioma tissue sampling for the optimal recurrence risk assessment.

**Abstract:**

The detection of the infiltrative growth of meningiomas into CNS tissue has been integrated into the WHO classification as a stand-alone marker for atypical meningioma. However, its prognostic impact has been questioned. Infiltrative growth can also be detected intraoperatively. The prognostic impact of the intraoperative detection of the central nervous system tissue invasion of meningiomas was analyzed and compared to the histopathological assessment. The clinical data of 1517 cases with follow-up data regarding radiographic recurrence was collected. Histopathology and operative reports were reviewed and invasive growth was seen during resection in 23.7% (*n* = 345) while histopathology detected it in 4.8% (*n* = 73). The histopathological and intraoperative assessments were compatible in 63%. The prognostic impact of histopathological and intraoperative assessment was significant in the univariate but not in the multivariate analysis. Both methods of assessment combined reached statistical significance in the multivariate analysis (*p* = 0.0409). A score including all independent prognostic factors divided the cohort into three prognostic subgroups with a risk of recurrence of 33.8, 64.7 and 88.5%, respectively. The intraoperative detection of the infiltrative growth of primary meningiomas into the central nervous system tissue can complement the histopathological assessment of CNS invasion. The combined assessment is an independent prognostic factor regarding tumor recurrence and allows a risk-adapted tumor stratification.

## 1. Introduction

With 37% of all intracranial tumors, meningioma is the most common primary central nervous system neoplasm [1] and can be treated effectively by surgical resection or in selected cases by radiotherapy [2]. However, approximately 20% show recurrence after 5 years [3]. Over time, different factors for the prediction of tumor recurrence were established. Histopathological signs of invasive growth into central nervous system tissue have been integrated into the WHO classification of meningiomas as a stand-alone criterion for atypia [4] while its prognostic value and difficulties of comprehensive assessment are frequently questioned and discussed [5,6,7]. The correct tumor sampling by the neurosurgeon is crucial for this assessment but not always possible due to direct contact or adhesions to critical neuronal structures. However, the intraoperative assessment regarding invasive growth into central nervous system tissue by the neurosurgeon might be of clinical value, especially in cases of incomplete sampling. The prognostic relation between intraoperative aspects of invasiveness and definite histopathological tissue assessment has not yet been evaluated in further detail. For this purpose, we retrospectively analyzed surgical and histopathological features of CNS invasion together with follow-up data in meningiomas regarding the risk of tumor recurrence.

## 2. Results

### 2.1. Cohort Characteristics

Out of 1741 cases, a total of 224 were lost to follow up (12.9%), leaving 1517 primary meningiomas for further analysis. Out of 1517 primary meningiomas, 1115 were from female and 402 from male patients (ratio 2.77). The mean age at diagnosis was 56.8 years, ranging from 3.8 to 89.9 years. The characteristics of the cohort with complete 5-year follow-up data (*n* = 550) were similar with a female to male ratio of 2.46 and a mean age of 54.6 years, ranging from 8.4 to 88.9. Furthermore, the distribution of tumor localization, the WHO grade and the extent of tumor resection (Simpson grade) are provided in Table 1, for the complete cohort and the 5-year follow-up cohort, respectively. To address a possible retrospective histopathological assessment bias, a subgroup analysis for cases treated after the implementation of the WHO classification of 2007 (*n* = 1216) was done with similar results (Table S1).

### 2.2. Univariate Analysis of Established Prognostic Factors

**Figure 1:**
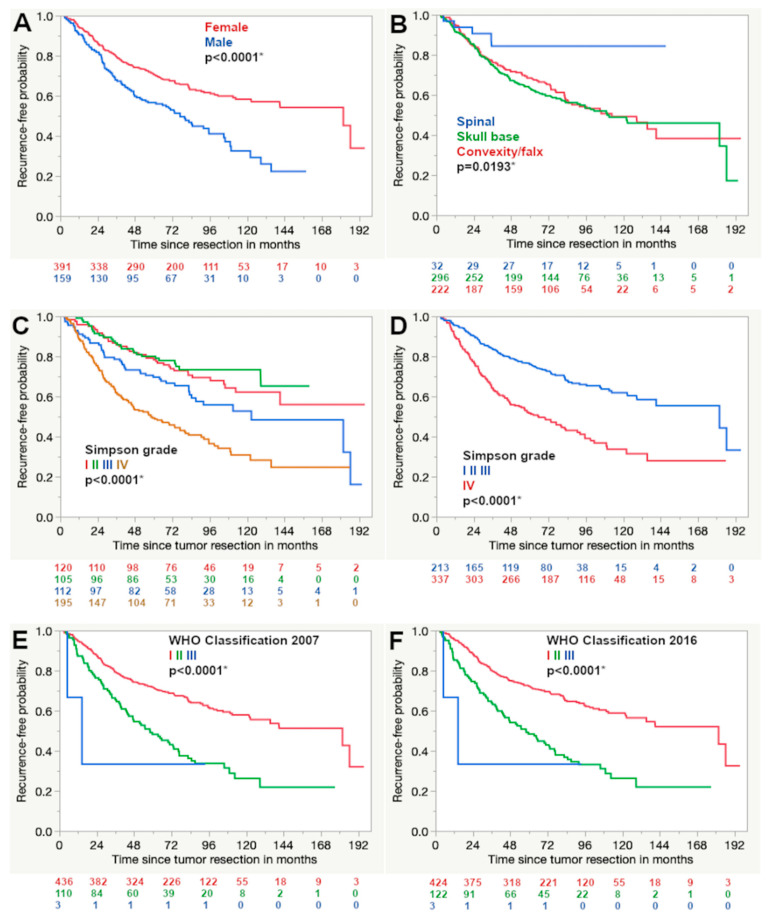
Kaplan–Meier curves show the results of the univariate survival analysis. A shorter progression-free survival was detected for male patients (**A**), non-spinal tumor location (**B**), higher Simpson (**C**,**D**) and WHO grade according to the classifications of 2007 and 2016 (**E**,**F**, respectively) (Log rank test, asterisk (*): statistically significant result).

Tumor recurrence or the growth of residual tumor was detected in 242 of 1517 cases (16.0%) while 1275 cases were documented as stable (84%) with a mean follow up of 39.6 months ranging from 1.2 to 195.6 months. Tumor recurrence was significantly more common in male patients (23.1% compared to 13.4%, *p* < 0.0001) and less frequently observed in spinal meningiomas when compared to the skull base and convexity/falx tumor localization (3.2% compared to 17.6% and 17.1%, respectively, *p* < 0.0001). A significantly lower rate of tumor recurrence was found for a higher extent of tumor resection according to the Simpson grading (*p* < 0.0001) as well as a lower WHO grading according to the classifications of 2007 (*p* < 0.0001) and 2016 (*p* < 0.0001). Similar statistical significances were also seen in the corresponding Kaplan–Meier curves (Figure 1).

The level of statistical significance was also reached in the univariate analysis for all the aforementioned factors in the subcohort, with a follow up of 5 years or longer (Table 1) and in the subgroup analysis of cases treated after the implementation of the WHO classification of 2007 (Table S1).

### 2.3. Univariate Analysis of CNS Invasion

**Figure 2:**
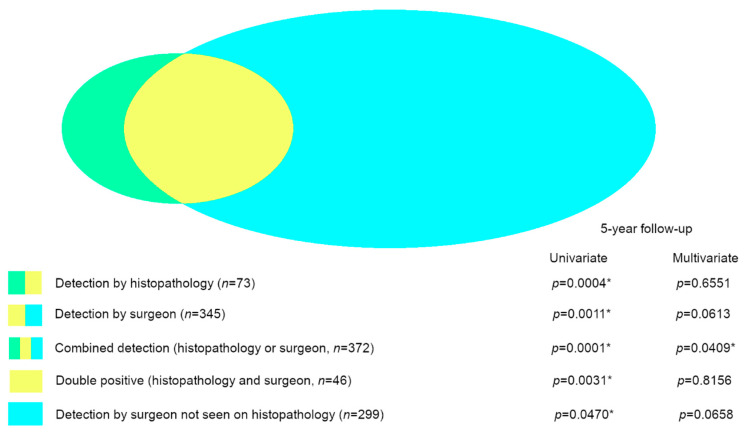
Graphical depiction of the different subgroups of primary meningiomas with CNS invasion that were analyzed. The color represents the mode of assessment: histopathology (green and yellow), intraoperative (yellow and blue), combined histopathology and intraoperative (green, yellow and blue), double positive by histopathology and intraoperative (yellow), only positive by intraoperative assessment (blue). All subgroups were significant factors for tumor recurrence in the univariate analysis, but only the combined assessment (histopathology and intraoperative) turned out to be an independent significant prognostic factor in the multivariate analysis (asterisk (*): statistically significant result).

By histopathological assessment, the infiltrative growth of meningioma cells into central nervous system tissue was detected in 73 of 1517 cases (4.8%). In comparison, clear intraoperative features of infiltrative growth were observed in 345 of 1455 cases (23.7%, operative report not available/inconclusive in 62 cases). The histopathological finding was compatible with the intraoperative assessment in 46 of 73 cases (63.0%) while 299 cases with a clear intraoperative infiltrative growth did not show CNS infiltration in the provided tissue sample for histopathological assessment (Figure 2).

**Figure 3:**
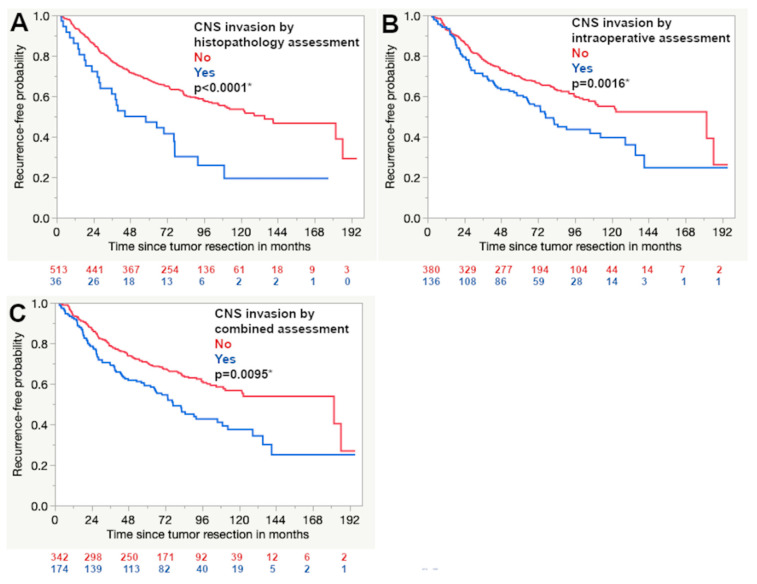
Kaplan–Meier curves demonstrate that the detection of CNS invasion by histopathology (**A**) and intraoperative assessment (**B**) were associated with a shorter progression-free survival. The combination of both detection methods was also a significant factor (**C**) (Log rank test, asterisk (*): statistically significant result).

The histopathological as well as the intraoperative detection of CNS invasion were associated with significantly higher rates of tumor recurrence (*p* < 0.0001 and 0.0002, respectively). This was also the case when all infiltrative meningiomas assessed as invasive by either intraoperative or histopathological measures were taken together (*n* = 372, *p* < 0.0001). Double-positive meningiomas that were rated as CNS invasive by both methods (histopathologically and intraoperatively assessed as infiltrative) showed a significantly higher recurrence rate as well (*n* = 46, *p* < 0.0001). For cases that were graded as CNS invasive only by intraoperative assessment (negative on histopathological assessment) statistical significance remained (*n* = 299, *p* = 0.0272). Significant results were also seen within the cohort with a follow-up of 5 years or longer (Table 1). Kaplan–Meier curves using the 5-year follow-up data showed significant Log-Rank test results as well (Figure 3).

Meningiomas with CNS invasion detected by histopathological, intraoperative or both methods show differences in gender, tumor location, WHO grade and histopathological subtype (according to the WHO classification of 2007). For example, while atypical meningiomas only make up 17.4% of invasive meningiomas detected by intraoperative assessment, there is a significantly higher portion among atypical tumors with invasion seen histopathologically (44.4%). If tumors were evaluated as invasive, both intraoperatively and histopathologically, 65.2% were classified as atypical meningiomas (Table S2). Similarly, in skull-base tumors, a higher frequency of CNS invasion was seen histologically (55.6%) compared to the intraoperative assessment (39.1%).

### 2.4. Multivariate Analysis

As the difference between the WHO classifications for meningiomas of 2007 and 2016 was the integration of CNS invasion in grade tumors as atypical, we used the WHO classification of 2007 to assess the prognostic impact of CNS invasion detected by histopathological and intraoperative assessment. All factors that showed significant prognostic results in the univariate analysis were integrated into the multivariate analysis. In the first model, gender, tumor localization (skull base, convexity/falx and spinal), Simpson grade (dichotomized into Simpson grade < = 3 and >3) and WHO grade according to the WHO classification of 2007 were analyzed together with CNS invasion by histopathological assessment (Figure 2, Table 2). Higher WHO and Simpson grade were independent negative prognostic factors (each *p* < 0.0001), as well as male gender (*p* = 0.0227). Tumor localization and histopathologically detected CNS invasion did not show an independent significant prognostic impact (*p* = 0.0562 and *p* = 0.6551, respectively).

When regarding the intraoperative detection of CNS invasion alone, the results of the multivariate analysis were similar (Figure 2, Table 2). Again, the WHO grade, Simpson grade and gender were significant independent predictors of tumor recurrence (*p* < 0.0001, *p* < 0.0001 and *p* = 0.04474, respectively), while tumor localization and the intraoperative detection of CNS invasion failed statistical significance (*p* = 0.0803 and *p* = 0.0613, respectively). The details of the analysis are shown in Table 2.

Subsequently, we tested the combined histopathological and intraoperative assessment of CNS invasion in the multivariate analysis and this resulted in statistical significance and confirmed CNS invasion as an independent negative prognostic factor (*p* = 0.0409). While the WHO and Simpson grade remained strong independent prognostic factors (each *p* < 0.0001), tumor localization and gender were without significant impact (*p* = 0.0862 and *p* = 0.0541, respectively) (Figure 2, Table 3).

### 2.5. Predictive Score

**Figure 4:**
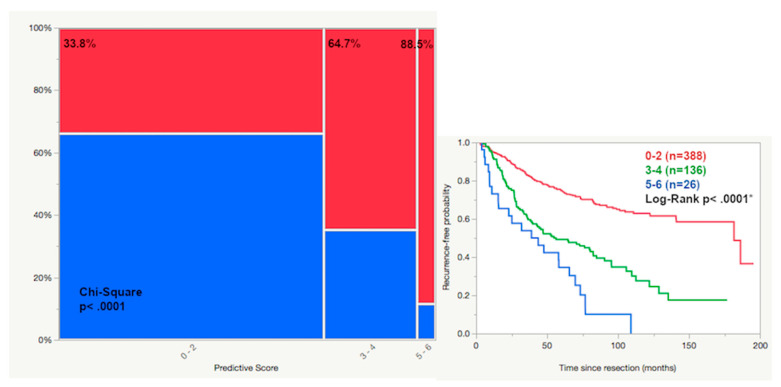
The predictive score consists of the sum of the following factors: male gender (1), CNS invasion detected by histopathology or intraoperative assessment (1), WHO grade II or III (2) and Simpson grade >3 (2). Scoring groups of 0–2, 3–4 and 5–6 were generated and showed a significant difference in tumor recurrence of 33.8, 64.7 and 88.5%, respectively (recurrence red, non-recurrence blue, 5-year follow-up or longer, asterisk (*): statistically significant result).

Based on the uni- and multivariate prognostic results, we generated a predictive score for the risk assessment using the rounded β-coefficient for each score. CNS invasion by combined assessment (histopathological and intraoperative) and male gender were given 1 point each, while WHO grade II or III (according to the classification of 2007) and Simpson grade >3 were given 2 points each, resulting in a predictive score of 0 to 6. When the score was applied to the cohort with a follow up of at least 5 years, 388 patients reached a score of 0 to 2 with 33.8% suffering a tumor recurrence. A score of 3 or 4 was given to 136 patients with a recurrence rate of 64.7%, while a score of 5 or 6 in 26 cases increased the rate of recurrence to 88.5% (significance across groups: *p* < 0.0001, see Figure 4).

## 3. Discussion

Since the latest update of the WHO classification for central nervous system tumors the histopathological detection of CNS invasion is a stand-alone criterion for atypical meningiomas, even in absence of cell atypia or mitotic activity [4]. However, its prognostic impact has been questioned [5]. Biczok et al. analyzed a cohort of 875 meningiomas of two neurosurgical institutions. Special emphasis was put on a subgroup where the meningioma–brain interface was available for histopathological assessment. Nonetheless, the authors did not find a prognostic impact of CNS invasion [8]. Another retrospective study assessed CNS invasion in 229 meningiomas and did not find a significantly increased risk of recurrence, claiming that especially otherwise benign meningiomas are unlikely to recur despite the presence of CNS invasion [9]. For this reason, we conducted a retrospective analysis with a high number of cases and a differentiation between histopathological and intraoperative assessment. In our study cohort, histopathologically assessed CNS invasion was a significant negative prognostic factor in the univariate analysis, but failed statistical significance in the multivariate analysis, thus confirming its prognostic uncertainty expressed in prior publications. However, by combining the histopathological and intraoperative assessment, CNS invasion was revealed as a significant independent prognostic factor in our cohort of primary meningiomas.

This result emphasizes the main limitation of the histopathological assessment of CNS invasion: non-standardized intraoperative tumor sampling. Recently, Timme et al. described how the frequency of brain invasion in meningiomas among different neurosurgical centers differed, suggesting an impact of surgical technique on tumor sampling. They showed that samples including brain tissue were more frequently observed in convexity/falx meningiomas [7]. Adequate tissue sampling from all areas with a meningioma–CNS interface is crucial to allow for the proper histopathological assessment of infiltrative growth [10]. In a systematic review, Brokinkel et al. found a higher rate of CNS invasion if the presence of CNS tissue was mandatory for histopathological evaluation [5]. Unfortunately, this is not always surgically feasible depending on the tumor location and the necessary surgical technique with the highest patient safety. It is clear that in such cases, the histopathological detection of CNS invasion may be impossible and the intraoperative assessment by the neurosurgeon can be of great value. On the other hand, the intraoperative assessment is clearly prone to interobserver variance depending on the surgeons’ experience, which is the main limitation of this retrospective analysis. Therefore, it is important to establish clear criteria for the intraoperative assessment of CNS invasion. For example, one aspect could be a separate sampling of areas with intraoperative aspects of infiltration or even frozen sections that could not only improve the histopathological yield but also potentially guide the resection technique. Our retrospective analysis serves as a starting point. A combined assessment has the potential to overcome the limitations of sole histopathological assessment as shown by the independent prognostic impact of combined histopathological and intraoperative assessments in our cohort. This underlines the necessity to combine our interdisciplinary efforts in daily practice.

One of the most striking results of our analysis is the large difference of CNS invasion detected by histopathology (73/1517, 4.8%) and intraoperative assessment (n = 345/1455, 23.7%). Even though 63% of histopathological CNS invasion was also seen intraoperatively, a large number of cases were rated as infiltrative, intraoperatively and non-invasive histopathologically (n = 299), most likely due to absence of CNS tissue in the sampled specimen. A discrepancy between the surgical and histologic assessment has been described before [6]. It is clear that the intraoperative assessment is not directly comparable with the histopathological detection. Even though under high magnification the breaching of the pial and arachnoid surface by meningioma tissue is detectable, a clear histopathological visualization of meningioma tissue protruding into the CNS is superior. Nonetheless, the issue of undersampling and the biased intraoperative assessment have to be addressed. The introduction of a standardized postoperative surgical evaluation sheet which demands clear statements on the arachnoid/pial breach, incomplete sampling and exact Simpson grading. In cases of complete removal and the sampling of the whole meningioma–CNS interface, the neuropathologist should have no reservations to attest non-invasiveness even if no CNS tissue is seen on the histological evaluation. It is recommended that neuropathological reports also contain a statement regarding the absence of CNS tissue in the samples. The data from the evaluation sheet could be used for a six-tiered risk score in conjunction with the histological findings.

In addition, the further improvement of recurrence prediction in meningioma patients may be achieved by the integration of preoperative imaging, since increasing evidence suggests its prognostic potential in detecting CNS invasion and recurrence [11,12]. An interdisciplinary collaborative retrospective analysis from our institution has also expressed the need for an integrated assessment of the brain and bone invasion of meningiomas and its prognostic potential [13].

An established factor that is associated with a higher risk of meningioma progression and recurrence is the expression of the proliferation marker MIB1. Its prognostic impact and correlation with volumetric growth has been shown in numerous studies [14] and it is an integral part of the routine neuropathological workup for meningiomas [4]. Although, the expression of MIB1 has been analyzed in this study indirectly by the inclusion of the WHO grade of each tumor, a detailed analysis of the correlation of MIB1 expression and invasive growth in future studies would be of great interest.

Furthermore, the integration of innovative techniques into the operating room that visualize invasive growth could allow for an objective intraoperative assessment. The methodology of intraoperative fluorescein imaging and the visualization of 5-aminolevulinic acid (5-ALA)-fluorescence show potential for intraoperative use [15,16,17].

This study underlines the need for an integrated approach and its potential. Especially in the light of the updated WHO classification for meningiomas and future molecular stratifications, it is crucial to combine our interdisciplinary efforts in order to improve treatment decision making for meningioma patients.

## 4. Materials and Methods

**Figure 5:**
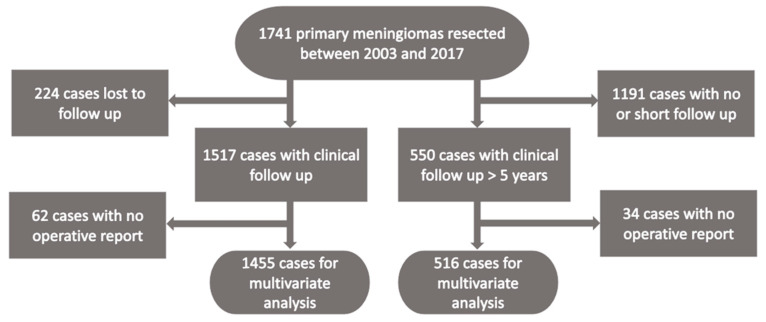
Flow chart of the study cohort.

In this retrospective translational single center study, we analyzed the prognostic effect of central nervous system invasion in meningiomas according to histopathological tissue analysis and intraoperative assessment by the neurosurgeon. Between October 2003 and March 2017, a total of 1741 primary meningiomas were surgically resected in the authors’ institution (Figure 5). Provided tissue was processed into formalin-fixed paraffin-embedded (FFPE) specimens in the department of Neuropathology. Histology slides were assessed for signs of invasive growth into central nervous system tissue by two neuropathologists independent of tumor grading. Brain invasion was diagnosed according to the criteria described by Perry et al. [6]. If no clear statement was documented in the neuropathological report, the tumor was graded as having no invasive features. Operative reports were reviewed for clear statements on invasive tumor growth or breach of the pial–arachnoid border. If no clear statement was made, the tumor was graded as having no intraoperative invasive features. The following clinical data were collected: age, gender, histopathological diagnosis, extent of resection (Simpson grade), tumor location and time to radiographic tumor recurrence/progression. For cases with a missing operative report, other documents were reviewed regarding the extent of resection if a clear statement on the Simpson grade was documented. Tumor localization was classified into a skull base, convexity/falx and spinal, while the Simpson grade was used in its classical grading from 1 to 5 [18] and for multivariate analysis in a dichotomized form. Samples were classified according to both WHO classifications for central nervous system tumors of 2007 (excluding CNS invasion as atypia criterion) and of 2016 (including histological invasion as criterion for atypia) [4].

The study was approved by the Clinical Ethics Committee of the University of Tübingen (Project number 618/2014BO2).

The primary outcome was tumor recurrence. All variables that showed a significant prognostic impact in the univariate analysis were integrated in the multivariate analysis. Additionally, all subgroups of CNS invasion were integrated as well to assess whether an independent prognostic impact was present. Statistical analysis was done with JMP^®^ Statistical Discovery Software, version 15.1.0 (Cary, NC: SAS Institute Inc.; 1989). The chi-square and Log-Rank test were used for the univariate, and the Wald test for the multivariate cox proportional hazard model while a significance level of α < 0.05 was applied. To generate the prognostic score, the β-coefficient of the risk models were used for all factors that reached statistical significance in the multivariate analysis and a score of 1 or 2 was chosen for each factor according to the rounded β-coefficient.

## 5. Conclusions

The detection of the infiltrative growth of primary meningiomas into the central nervous system tissue differs between intraoperative and histopathological assessment. Each method has its limitations of prognostication. The combined assessment is an independent prognostic factor regarding tumor recurrence. However, it remains crucial to mind the correct tissue sampling of all areas adjacent to central nervous system tissue to allow optimal histopathological yield of infiltrative growth assessment.

## Figures and Tables

**Table 1:** Cohort characteristics and univariate analysis of tumor recurrence (chi-square test).

Variable	Complete Cohort				5-Year Follow-Up Cohort			
	N (%)	Tumor Recurrence n (%)		*p*-Value	N (%)	Tumor Recurrence n (%)		*p*-Value
		Yes	No			Yes	No	
Gender								
Female	1115 (73.5)	149 (13.4)	966 (86.6)	<0.0001 *	391 (71.1)	149 (38.1)	242 (61.9)	<0.0001 *
Male	402 (26.5)	93 (23.1)	309 (76.9)		159 (28.9)	93 (58.5)	66 (41.5)	
Tumor localization								
Skull base	788 (51.9)	139 (17.6)	649 (82.4)	<0.0001 *	296 (53.8)	139 (47.0)	157 (53.0)	0.0032 *
Convexity/falx	574 (37.8)	98 (17.1)	476 (82.9)		222 (40.4)	98 (44.1)	124 (55.9)	
Spinal	155 (10.2)	5 (3.2)	150 (96.8)		32 (5.8)	5 (15.6)	27 (84.4)	
Simpson grade								
1	366 (24.8)	40 (10.9)	326 (89.1)	<0.0001 *	120 (22.6)	40 (33.3)	80 (66.7)	<0.0001 *
2	411 (27.8)	27 (6.6)	384 (93.4)		105 (19.7)	27 (25.7)	78 (74.3)	
3	308 (20.8)	48 (15.6)	260 (84.4)		112 (21.1)	48 (42.9)	64 (57.1)	
4	393 (26.6)	122 (31.0)	271 (69.0)		195 (36.7)	122 (62.6)	73 (37.4)	
5	0 (-)	0 (-)	0 (-)		0 (-)	0 (-)	0 (-)	
CNS invasion by histopathological assessment alone								
Yes	73 (4.8)	26 (35.6)	47 (64.4)	<0.0001 *	36 (6.6)	26 (72.2)	10 (27.8)	0.0004 *
No	1444 (95.2)	216 (15.0)	1228 (85.0)		514 (93.5)	216 (42.0)	298 (58.0)	
CNS invasion by intraoperative assessment alone								
Yes	345 (23.7)	76 (22.0)	269 (78.0)	0.0002 *	136 (26.4)	76 (55.9)	60 (44.1)	0.0011 *
No	1110 (76.3)	151 (13.6)	959 (86.4)	380 (73.6)	151 (39.7)		229 (60.3)	
CNS invasion by combined assessment								
Yes	372 (25.6)	85 (22.9)	287 (77.2)	<0.0001 *	149 (28.9)	85 (57.1)	64 (43.0)	0.0001 *
No	1083 (74.4)	142 (13.1)	941 (86.9)		367 (71.1)	142 (38.7)	225 (61.3)	
CNS invasion intraoperatively not seen on histopathology								
Yes	299 (20.6)	59 (19.7)	240 (80.3)	0.0272 *	113 (21.9)	59 (52.2)	54 (47.8)	0.0464 *
No	1156 (79.5)	168 (14.5)	988 (85.5)		403 (78.1)	168 (41.7)	235 (58.3)	
CNS invasion double positive (histopathology and intraoperatively)								
Yes	46 (3.2)	17 (37.0)	29 (63.0)	<0.0001 *	23 (4.5)	17 (73.9)	6 (26.1)	0.0031 *
No	1409 (96.8)	210 (14.9)	1199 (85.1)		493 (95.5)	210 (42.6)	283 (57.4)	
WHO classification 2007								
I	1313 (86.6)	166 (12.6)	1147 (87.4)	<0.0001 *	437 (79.5)	166 (38.0)	271 (62.0)	<0.0001 *
II	200 (13.2)	74 (37)	126 (63)		110 (20.0)	74 (67.3)	36 (32.7)	
III	4 (0.3)	2 (50)	2 (50)		3 (0.6)	2 (66.7)	1 (33.3)	
WHO classification 2016								
I	1281 (84.4)	158 (12.3)	1123 (87.7)	<0.0001 *	425 (77.3)	158 (37.2)	267 (62.8)	<0.0001 *
II	232 (15.3)	82 (35.3)	150 (64.7)		122 (22.2)	82 (67.2)	40 (32.8)	
III	4 (0.3)	2 (50)	2 (50)		3 (0.56)	2 (66.7)	1 (33.3)	

Asterisk (*): statistically significant result.

**Table 2:** Multivariate analysis of the histopathologically and intraoperatively assessed CNS invasion (Cox proportional hazard).

Variable	Histopathology		Intraoperative	
	Risk Ratio (95% CI)	*p*-Value (Prob > Chisq)	Risk Ratio (95% CI)	*p*-Value (Prob > Chisq)
Male gender	1.38 (1.05–1.82)	0.0227 *	1.33 (1.00–1.77)	0.0474 *
Localization				
Spinal vs. skull base	0.42 (0.17–1.02)	0.0562	0.45 (0.18–1.10)	0.0803
Spinal vs. convexity/falx	0.56 (0.22–1.38)	0.2084	0.62 (0.24–1.54)	0.3009
Convexity/falx vs. skull base	0.75 (0.56–0.99)	0.0430 *	0.73 (0.54–0.99)	0.0411 *
Simpson grade </= 3	0.42 (0.32–0.55)	<0.0001 *	0.40 (0.30–0.52)	<0.0001 *
WHO classification 2007				
I vs. II	0.40 (0.29–0.56)	<0.0001 *	0.44 (0.32–0.60)	<0.0001 *
I vs. III	0.19 (0.05–0.80)	0.0239 *	0.17 (0.04–0.73)	0.0169 *
II vs. III	0.48 (0.12–1.99)	0.311	0.40 (0.10–1.66)	0.2062
CNS invasion by histopathological/intraoperative assessment	1.11 (0.70–1.76)	0.6551	1.34 (0.99–1.82)	0.0613

Asterisk (*): statistically significant result.

**Table 3:** Multivariate analysis of the combined histopathological and intraoperative assessment of CNS invasion (Cox proportional hazard).

Variable	Risk Ratio (95%CI)	*p*-Value (Prob > Chisq)
Male gender	1.32 (1.00–1.75)	0.0541
Localization		
Spinal vs. skull base	0.45 (0.19–1.12)	0.0862
Spinal vs. convexity/falx	0.62 (0.24–1.55)	0.3022
Convexity/falx vs. skull base	0.74 (0.55–1.00)	0.0447 *
Simpson grade </= 3	0.40 (0.31–0.53)	<0.0001 *
WHO classification 2007		
I vs. II	0.45 (0.33–0.63)	<0.0001 *
I vs. III	0.17 (0.04–0.73)	0.0167 *
II vs. III	0.38 (0.09–1.61)	0.1894
CNS invasion by combined assessment (histology and intraoperatively)	1.37 (1.01–1.85)	0.0409 *

Asterisk (*): statistically significant result.

## Supplementary Materials

The following are available online at https://www.mdpi.com/2072-6694/12/12/3620/s1, Table S1: Cohort characteristics and univariate analysis of tumor recurrence restricted to cases treated after the implementation of the WHO classification of 2007 (Chi-Square test), Table S2: Characteristics of meningiomas with invasive growth according to the type of assessment (Chi-Square test).
