# Supplementary material for: CNS Invasion in Meningioma—How the Intraoperative Assessment Can Improve the Prognostic Evaluation of Tumor Recurrence
Source: Cancers (Basel). 2020 Dec 3;12(12):3620. doi: 10.3390/cancers12123620 (PMC7761660; doi:10.3390/cancers12123620)
Supplement: Supplementary file 1 [file cancers-12-03620-s001.pdf]

Supplementary Materials: CNS Invasion in Meningioma—How the Intraoperative Assessment Can Improve the Prognostic Evaluation of Tumor Recurrence

Felix Behling, Christina Fodi, Irina Gepfner-Tuma, Kathrin Machetanz, Mirjam Renovanz, Marco Skardelly, Antje Bornemann, Jürgen Honegger, Ghazaleh Tabatabai, Marcos Tatagiba and Jens Schittenhelm

Table S1. Cohort characteristics and univariate analysis of tumor recurrence restricted to cases treated after the implementation of the WHO classification of 2007 (Chi-Square test).

**Variable**	**Complete Cohort**				**5-year Follow-up Cohort**			
	**N (%)**	**Tumor Recurrence N (%)**		***p*-value**	**N (%)**	**Tumor Recurrence N (%)**		***p*-value**
		Yes	No			Yes	No	
Gender								
Female	895 (73.6)	114 (12.7)	781 (87.3)	.0001*	277 (70.7)	114 (41.2)	163 (58.9)	.0004*
Male	321 (26.4)	70 (21.8)	251 (78.2)		115 (29.3)	70 (60.9)	45 (39.1)	
Tumor localization								
Skull base	633 (52.1)	109 (17.2)	524 (82.8)	.0007*	212 (54.1)	109 (51.4)	103 (48.6)	.0140*
Convexity/falx	456 (37.5)	70 (15.4)	386 (84.7)		156 (39.8)	70 (44.9)	86 (55.1)	
Spinal	127 (10.4)	5 (3.9)	122 (96.1)		24 (6.1)	5 (20.8)	19 (79.2)	
Simpson grade								
1	286 (24.1)	24 (8.4)	262 (91.6)	< .0001*	76 (20.0)	24 (31.6)	52 (68.4)	< .0001*
2	332 (28.0)	19 (5.7)	313 (94.3)		72 (19.0)	19 (26.4)	53 (73.6)	
3	248 (20.9)	39 (15.7)	209 (84.3)		84 (22.1)	39 (46.4)	45 (53.6)	
4	319 (26.9)	99 (31.0)	220 (69.0)		148 (39.0)	99 (66.9)	49 (33.1)	
5	0 (-)	0 (-)	0 (-)		0 (−)	0 (−)	0 (−)	
CNS invasion by histopathological assessment								
Yes	63 (5.2)	21 (33.3)	42 (66.7)	< .0001*	29 (7.4)	21 (72.4)	8 (27.6)	.0043*
No	1153 (94.8)	163 (14.1)	990 (85.9)		363 (92.6)	163 (44.9)	200 (55.1)	
CNS invasion by intraoperative assessment								
Yes	275 (23.5)	51 (18.6)	224 (81.5)	.0454*	96 (26.0)	51 (53.1)	45 (46.9)	.1542
No	894 (76.5)	122 (13.7)	772 (86.4)		273 (74.0)	122 (44.7)	151 (55.3)	
CNS invasion by combined assessment								
Yes	301 (25.8)	60 (19.9)	241 (80.1)	.0036*	108 (29.3)	60 (55.6)	48 (44.4)	.0318*
No	868 (74.3)	113 (13.0)	755 (87.0)		261 (70.7)	113 (43.3)	148 (56.7)	
CNS invasion intraoperatively not seen on histopathology	238 (20.4)	39 (16.4)	199 (83.6)	.4396	79 (21.4)	39 (49.4)	40 (50.6)	.6178
Yes	931 (76.6)	134 (14.4)	797 (85.6)		290 (78.6)	134 (46.2)	156 (53.8)	
No								
CNS invasion double positive (histopathology and	37 (3.2)	12 (32.4)	25 (67.6)	.0021*	17 (4.6)	12 (70.6)	5 (29.4)	.0449*
intraoperatively)	1132 (96.8)	161 (14.2)	971 (85.8)		352 (95.4)	161 (45.7)	191 (54.3)	

Yes								
No								
WHO Classification	1066 (87.7)	129 (12.1)	937 (87.9)	< .0001*	311 (79.3)	129 (41.5)	182 (58.5)	<.0001*
2007	147 (12.1)	53 (36.1)	94 (64)		79 (20.2)	53 (67.1)	26 (32.9)	
I	3 (0.3)	2 (66.7)	1 (33.3)		2 (0.5)	2 (100.0)	0 (0)	
II								
III								
WHO Classification	1035 (85.1)	122 (11.8)	913 (88.2)	< .0001*	300 (76.5)	122 (40.7)	178 (59.3)	<.0001*
2016	178 (14.6)	60 (33.7)	118 (66.3)		90 (23.0	60 (66.7)	30 (33.3)	
I	3 (0.3)	2 (66.7)	1 (33.3)		2 (0.5)	2 (100.0)	0 (0)	
II								
III								

Asterisk (*): statistically significant result.

Table S2. Characteristics of meningiomas with invasive growth according to the type of assessment (Chi-Square test).

	N (%)	CNS Invasion			*p*-value
		**Histopathology Assessment**	**Intraoperative Assessment**	**By Both Assessments**	
Gender					
Female	245 (65.9)	11 (40.7)	208 (69.6)	26 (56.5)	
Male	127 (34.1)	16 (59.3)	91 (30.4)	20 (43.5)	
Tumor localization					
Skull base	142 (38.2)	15 (55.6)	117 (39.1)	10 (21.7)	
Convexity/falx	226 (60.8)	12 (44.4)	178 (59.5)	36 (78.3)	0.0037*
Spinal	4 (1.1)	0 (−)	4 (1.3)	0 (−)	
WHO classification					
2007					0.0402*
I	271 (72.9)	15 (55.60	240 (80.3)	16 (34.8)	
II	100 (26.9)	12 (44.4)	58 (19.4)	30 (65.2)	
III	1 (0.3)	0 (−)	1 (0.3)	0 (−)	
Histology					
I					<0.0001*
Angiomatous	7 (1.9)	0 (−)	7 (2.3)	0 (−)	
Fibroblastic	17 (4.6)	0 (−)	16 (5.4)	1 (2.2)	
Lymphocyte rich	0 (−)	0 (−)	0 (−)	0 (−)	
Meningothelial	157 (42.2)	4 (14.8)	144 (48.2)	9 (19.6)	
Metaplastic	5 (1.3)	1 (3.7)	4 (1.3)	0 (−)	<0.0001*
Microcystic	11 (3.0)	0 (−)	11 (3.7)	0 (−)	
Psammomatous	9 (2.4)	0 (−)	9 (3.0)	0 (−)	
Secretory	16 (4.3)	2 (7.4)	13 (4.4)	1 (2.2)	
Transitional	32 (8.6)	1 (3.7)	31 (10.4)	0 (−)	
NOS	17 (4.6)	7 (25.9)	5 (1.7)	5 (10.9)	
II					
Atypical	94 (25.3)	12 (44.4)	52 (17.4)	30 (65.2)	
Chordoid	6 (1.6)	0 (−)	6 (2.0)	0 (−)	
Clear Cell	0 (−)	0 (−)	0 (−)	0 (−)	
III					
Anaplastic	0 (−)	0 (−)	0 (−)	0 (−)	
Papillary	0 (−)	0 (−)	0 (−)	0 (−)	
Rhabdoid	1 (0.3)	0 (−)	1 (0.3)	0 (−)	

Asterisk (*): statistically significant result.

© 2020 by the authors. Licensee MDPI, Basel, Switzerland. This article is an open access article distributed under the terms and conditions of the Creative Commons Attribution (CC BY) license (http://creativecommons.org/licenses/by/4.0/).
